# Effect of Comb Cell Width on the Activity of the Proteolytic System in the Hemolymph of *Apis mellifera* Workers

**DOI:** 10.3390/ani12080978

**Published:** 2022-04-10

**Authors:** Piotr Dziechciarz, Aneta Strachecka, Krzysztof Olszewski

**Affiliations:** 1 Department of Apidology, Institute of Biological Basis of Animal Production, Faculty of Animal Sciences and Bioeconomy, University of Life Sciences in Lublin, 20-950 Lublin, Poland; krzysztof.olszewski@up.lublin.pl; 2 Department of Invertebrate Ecophysiology and Experimental Biology, University of Life Sciences in Lublin, 20-950 Lublin, Poland; aneta.strachecka@up.lublin.pl

**Keywords:** *Apis mellifera*, hemolymph, proteolytic system, proteases, protease inhibitors, bee comb, small-cell combs

## Abstract

**Simple Summary:**

Honeybees are threatened by many biotic factors, e.g., microbes and parasites such as *Nosema* spp. and *Varroa* mites, or predators, as well as environmental factors such as weather conditions, pesticides, or food contaminants. Honeybee colonies have behavioral defense mechanisms against pathogens resulting from their social lifestyle. Moreover, these insects have innate immune barriers. One of the immune defense elements is the proteolytic system consisting of proteases and their inhibitors in the hemolymph (insect blood) and on the body surface. The proteolytic system is activated by both external and internal environmental factors. An important element of the nest environment is the comb. In feral bee colony nests, the bee comb cell size varies in width. In turn, bee colonies in Europe tend to be kept only on standard- (cell width approx. 5.50 mm) or small-cell (cell width approx. 4.90 mm) combs. We assessed the proteolytic system activity in the hemolymph of workers reared in a small-cell comb and a standard-cell comb in colonies kept simultaneously on standard- and small-cell combs. Simultaneous keeping of a colony on standard- and small-cell combs is a novel approach to the use of small-cell combs in beekeeping. The width of comb cells (small or standard) where workers were reared had a significant effect on the protein concentrations and the activities of proteases and protease inhibitors in hemolymph of workers. The protein concentrations in 1-day-old workers were higher in bees reared in small-cell combs than in those reared in standard-cell combs. The opposite was found in the groups of older bees (aged 7, 14 and 21 d). Moreover, the activities of proteases and their inhibitors in 1-day-old workers were always higher in bees reared in standard-cell combs, whereas opposite results were usually obtained in the group of the older workers. The differences between workers reared in the small-cell combs and those from the standard-cell combs may be associated with their different tasks. Workers reared in small-cell combs probably work outside the nest as foragers, whereas those reared in standard-cell combs work in the nest. However, this assumption requires confirmation.

**Abstract:**

This study is a continuation of the innovative research of the impact of rearing of bee colonies simultaneously on standard- and small-cell combs on the traits of worker bees and bee colonies. Its aim was to compare the activities of proteases and their inhibitors in the hemolymph of workers reared in a small-cell comb (SMC) and a standard-cell comb (STC) in colonies kept simultaneously on standard- and small-cell combs. The width of comb cells in which workers are reared has a significant effect on the protein concentration and the proteolytic system in the hemolymph, which is reflected in the activities of proteases and their inhibitors. The protein concentrations in the 1-day-old workers were always higher (*p* ≤ 0.05) in the SMC than STC workers. The opposite was found in the older bee workers (aged 7, 14 and 21 d). The activities of proteases and their inhibitors in the 1-day-old workers were always higher (usually significantly at *p* ≤ 0.05) in STC than SMC workers, and opposite results were observed in the groups of the older workers (aged 7, 14 and 21 d). The differences between the workers from small-cell combs and those reared in standard-cell combs may be related to their different tasks. Workers reared in small-cell combs probably work as foragers outside the nest, whereas bees reared in standard-cell combs work in the nest. This hypothesis requires confirmation. To reduce the impact of accidental determinants on the results of single-season research on honeybees, it is advisable that such investigations should be conducted for several consecutive years.

## 1. Introduction

The diversity of ecological niches inhabited by insects poses a potential threat to bees through the presence of multiple biotic factors, e.g., microbes and parasites such as *Nosema* spp. and *Varroa* mites, or predators, as well as environmental factors such as weather conditions, pesticides, or food contaminants. [1]. During flight, foragers are permanently exposed to external environmental factors; additionally, the environment in the bee colony nest itself may carry a risk of the presence of a pathogenic agent due to the concentration of a large number of individuals in a small space [2]. Honeybees have developed group/behavioral mechanisms of active disruption of the disease chain in the colony, e.g., grooming behavior [3], colony fever [4], and hygienic behavior [5,6,7]. In addition, bees have innate immunological barriers against pathogens [8].

Immune proteins fulfill their functions thanks to the coordinated interaction of the fat body in which they are synthesized and the hemolymph through which they are transported. In addition, the hemolymph provides an appropriate environment for these proteins to function. Therefore, it is also a source of information about the physiological state of the organism [9]. Some of these proteins have proteolytic properties, i.e., they can hydrolyze (cut) peptide bonds of polypeptides [8,10]. Proteases facilitate maintenance of homeostasis in the organism through activation of proenzymes, release of hormones and physiologically active proteins from their precursors, or activation of receptors. They also target the enzymatic breakdown of pathogens’ proteins [11,12,13,14]. The hemolymph of bees contains serine, asparagine, cysteine proteases, and metalloproteases [15,16]. In turn, protease inhibitors inhibit the activities of bee-specific and pathogen-specific proteolytic enzymes and prevent pathogens from penetration into the insect’s body. In addition, they protect against accidental activation of mechanisms related to the activity of phenyloxidase, which is involved in such processes as metamorphosis and melanization [10,17].

The activity of the proteolytic system, which is one of the measures of humoral immunity in the bee hemolymph, is influenced by pesticides [18,19,20], acaricides used against *Varroa destructor* [21], biostimulants [22,23], and caste status [24].

Bees have no impact on external conditions that directly involve their immune system, but in response they are capable of precise regulation of the conditions prevailing inside their nest. One of the elements responsible for the regulation is the honeycomb, which is the basic element—an “organ” of the bee colony nest [25]. As a result of the introduction of an artificially produced wax foundation, comb cells built by bees have almost the same standardized width [26]. In Europe, the standard width of cells in the wax foundation is usually approx. 5.40–5.50 mm [26,27,28]. The width of the cells in natural combs built without the wax foundation has a substantially larger range, i.e., cells where worker brood is reared may be 4.17–6.86 mm wide [29].

At present, the use of the wax foundation is a standard practice in beekeeping management in developed countries. Nevertheless, the impact of the comb cell width on the traits of bees and bee colonies has been poorly explored so far. The scientific interest in small-cell combs began only in the 21st century after the publication of reports showing that rearing brood in small-cell combs versus standard-cell combs limits the growth of populations of the common bee parasite *V. destructor* [27,28,29,30,31,32,33,34]. In Europe, the width of small cells on the wax foundation is 4.90 mm [26,27,28]. In European conditions, the limitation of the *V. destructor* population through the use of small-cell combs was confirmed only by Singer et al. [28], and the attempts undertaken by beekeepers to keep colonies on small-cell combs probably did not bring the expected results, as these combs were never widely used. Nevertheless, a significant effect of the use of small-cell combs on the morphological traits of worker bees and bee colony biology was found. It consisted of a decrease in the thorax weight, head width and height, thorax width and length, width and length of fore wings, and width of the third and fourth tergites [26,34,35,36]. Researchers additionally reported a longer lifespan of workers reared in colonies kept on small cell combs [37] and the contribution of small-cell combs to the higher effectiveness of bees’ hygienic behavior [38].

We decided to investigate a new aspect of the use of small-cell combs in apiaries. Based on the research conducted by Maggi et al. [29], who reported a significant variation in the width of comb cells in a bee colony nest constructed without a wax foundation, we decided to combine small-cell combs and standard-cell combs in a nest of the same bee colony. The present study was prompted by our earlier research [36], where brood in small-cell combs was reared in a colony kept on standard-cell combs, i.e., by nurse workers reared in standard-cell combs. The values of the morphometric traits of the workers reared in the small-cell combs in a colony kept on standard-cell combs were higher than in bees reared in small-cell combs in a colony kept on small-cell combs. This indicates that the traits of bees are influenced not only by the width of the comb cells where they are reared, but also by the width of the comb cells where nurse workers were reared. It is highly probable that such a combination of combs with different cell widths in the same colony will also influence the traits of the entire colony as a complex superorganism. This conclusion was confirmed in our previous study [39], which showed a more effective removal of dead brood from small- than standard-cell combs in colonies kept simultaneously on standard- and small-cell combs.

This study is a continuation of our innovative research on the impact of simultaneous maintenance of bee colonies on standard- and small-cell combs on the traits of worker bees and bee colonies [36,39]. The aim of the paper was to compare the activities of proteases and their inhibitors in the hemolymph of workers reared in small-cell combs and standard-cell combs in colonies kept simultaneously on standard- and small-cell combs.

## 2. Materials and Methods

All the research procedures were conducted at the apiary of the University of Life Sciences in Lublin (51°22′ N, 22°63′ E). Environmental factors exert a considerable impact on honeybee traits [40]. Therefore, to reduce significantly the risk of an effect of random factors on the results that may occur in a single-season study, we repeated the experiment in the same design in three consecutive years: 2019, 2020 and 2021.

### 2.1. Acquisition of Bees

Each year, five foster colonies with similar strength and structure, headed by naturally mated Buckfast sister-queens of the same age, were used. We managed Buckfast bee colonies kept in our apiary, as they are very well adapted to living on small-cell combs [36,39]. All colonies were kept simultaneously on small-cell combs (cell width approx. 4.90 mm) and standard-cell combs (cell width approx. 5.50 mm) in Dadant Blatt hives. The scheme of the small- and standard-cell combs in the brood chamber was consistent with that presented by [39].

On the first d of June each year, in five foster colonies maintained simultaneously on the small- and standard-cell combs, we removed the standard-cell brood comb from the brood chamber and replaced it with a frame cage made of a queen excluder with an empty standard-cell experimental comb inside. The frame cage contained one comb. We placed and kept a queen in the frame cage for 24 h for oviposition in the experimental comb. After this time, we removed the small-cell brood comb from the brood chamber and replaced it with a standard-cell experimental comb from the frame cage with the eggs. The comb was labeled. We left the queen in the frame cage and placed an empty small-cell experimental comb. We kept the queen in the frame cage for another 24 h for oviposition in this comb. After this time, we removed the frame cage with the small-cell experimental comb and the queen as well as the standard-cell experimental comb removed earlier from the frame cage from the brood chamber. In the empty space in the brood chamber, we placed a frame cage containing two combs and placed both standard- and small-cell experimental combs with eggs. We placed the queen to the brood chamber and closed the frame cage to prevent the queen from entering the cage. The timing of the placement of the combs with eggs in the frame cage was not random. The small-cell experimental comb was placed a d later, as brood in such combs emerges one d earlier than brood in standard-cell combs. In this way, the imago stages emerged on the same d and similar-age workers were used in the experiment. No brood had been previously reared in any of the experimental combs.

After 20 d of keeping the queen in the frame cage on the standard-cell experimental comb, we placed each experimental comb in a separate mesh frame cage and kept them in an incubator until emergence of workers.

Approximately 1500 workers from the pool emerging from each experimental comb in each of the five foster colonies were labeled (POSCA PC-3M marker). Workers reared in the small-cell experimental comb (SMC) were labeled with a different color than those reared in the standard cell experimental comb (STC). We placed the labeled workers in five colonies kept in hives with six combs. The colonies had similar strength and structure; each had a properly ovipositing queen, five combs with different aged brood, and one comb with honey and bee breed. The queens in these colonies were sisters of the same age. The workers reared in each of the foster colonies were allocated to a separate colony. We used colonies kept on six combs, as it was easier to collect the labeled workers.

### 2.2. Collection of Hemolymph and Evaluation of the Activity of Proteases and Their Inhibitors in Bee Hemolymph

On d 7, 14 and 21 [21] after labeling the bees, SMC workers (reared in small-cell combs) and STC workers (reared in standard-cell combs) were selected randomly from each of the five colonies kept on the six combs. Hemolymph was collected from each worker [41]. One sample comprised hemolymph collected from five bees. The hemolymph obtained from each group of five workers was transferred into a separate Eppendorf tube (0.5 mL) filled with 150 µL of 0.6% NaCl and placed in a cooling block to prevent melanization. Hemolymph from the 1-day-old workers was collected on the labeling d. The number of samples taken in the consecutive years is shown in Table 1.

Immediately after hemolymph collection, the tubes were frozen and stored at −80 °C. Total protein concentrations were assayed with the Lowry et al. [42] method modified by Schacterle and Pollack [43]. The activities of acidic (pH 2.4), neutral (pH 7.0), and alkaline (pH 11.2) proteases in the hemolymph were analyzed with the Anson [44] method modified by Strachecka et al. [13]. Protease inhibitor activities were determined using Lee and Lin’s [45] method.

### 2.3. Values of the Characteristics of Weather Conditions

Values of the characteristics of weather conditions in years covering experimental period have been shown in Table 2. The data were obtained from the Lublin-Radawiec meteorological station.

### 2.4. Measurements of Comb Cell Width

Each experimental comb in which the workers were reared was photographed in the center of each comb half on one side of the comb. Next, in each half, the widths of 10 adjacent cells contacting with vertical side walls were measured following the procedure used by Dziechciarz et al. [36]. Each year (2019, 2020 and 2021), 100 cells (5 combs × 2 measurements of 10 cells per combs) were measured in each type of the experimental combs (small- and standard-cell comb).

### 2.5. Statistical Analysis

The statistical analysis of the results was processed using Statistica software formulas, version 13.3 (2017) for Windows, StatSoft Inc., Tulsa, OK, USA. The data distribution was analyzed with the use of the Shapiro–Wilk test.

The effect of the year on the hemolymph parameters (protein concentration and activities of acidic protease, neutral protease, alkaline protease, acidic protease inhibitors, neutral protease inhibitors, and alkaline protease inhibitors) was assessed separately for the SMC and STC workers using the Kruskal–Wallis test, as all these data had no normal distribution.

The effect of age on the hemolymph parameters (protein concentration and activities of acidic protease, neutral protease, alkaline protease, acidic protease inhibitors, neutral protease inhibitors, and alkaline protease inhibitors) was assessed in each year (2019, 2020 and 2021), separately for the SMC and STC workers with the use of the Kruskal–Wallis test, as all these data had no normal distribution.

The protein concentrations and activities of the proteases and their inhibitors in the hemolymph were compared in each year (2019, 2020, and 2021) and within each age group (1 d, 7 d, 14 d and 21 d) between the SMC and STC workers. The paired sample T test was used for normally distributed data, and data with no normal distribution were analyzed using the pairwise Wilcoxon test.

The relationships between the width of the experimental combs cell and the year (2019, 2020 and 2021) were assessed separately for the small-cell combs (*n* = 300) and the standard-cell combs (*n* = 300) with the Kruskal–Wallis test. The Mann–Whitney test was used to compare the cell width in the small-cell experimental combs with the cell width in the standard-cell combs in each year (2019, *n* = 100; 2020, *n* = 100; 2021, *n* = 100).

## 3. Results

### 3.1. Protein Concentration, Protease, and Protease Inhibitor Activities

In both the SMC and STC workers, the year (2019, 2020 and 2021) had a significant effect on all hemolymph parameters (Table 3).

In all years, the age (1 d, 7 d, 14 d and 21 d) exerted a significant effect on all hemolymph parameters in both the SMC and STC workers (Table 3).

The weather conditions in each experimental period in the subsequent years of research were similar (Table 2). Only the amount of rainfall in 2019 was much lower than in other years, and the time in which the experiment was conducted was preceded by a long drought.

**Figure 1:**
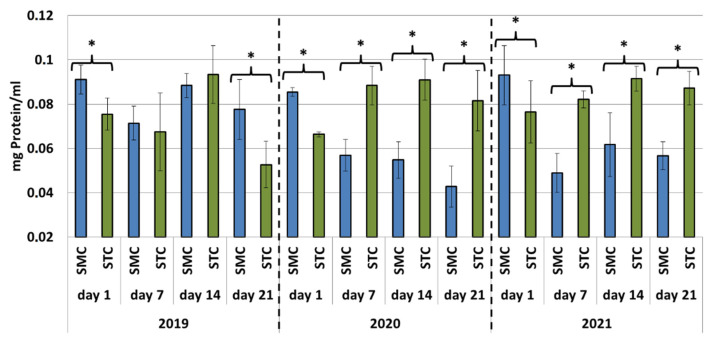
Protein concentrations in the hemolymph of workers in three consecutive years. SMC—workers reared in small-cell combs; STC—workers reared in standard-cell combs; *—differences between SMC and STC within the age group are significant at *p* ≤ 0.05.

**Figure 2:**
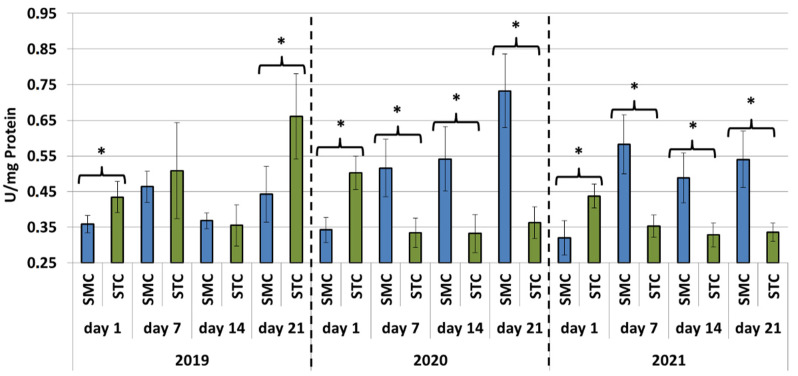
Acidic protease activities in the hemolymph of workers in three consecutive years. SMC—workers reared in small-cell combs; STC—workers reared in standard-cell combs; *—differences between SMC and STC within the age group are significant at *p* ≤ 0.05.

**Figure 3:**
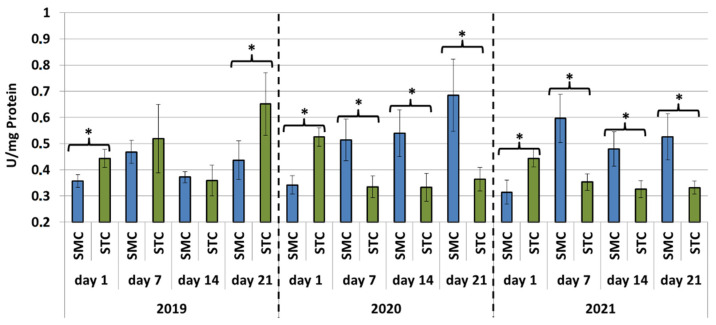
Neutral protease activities in the hemolymph of workers in three consecutive years. SMC—workers reared in small-cell combs; STC—workers reared in standard-cell combs; *—differences between SMC and STC within the age group are significant at *p* ≤ 0.05.

**Figure 4:**
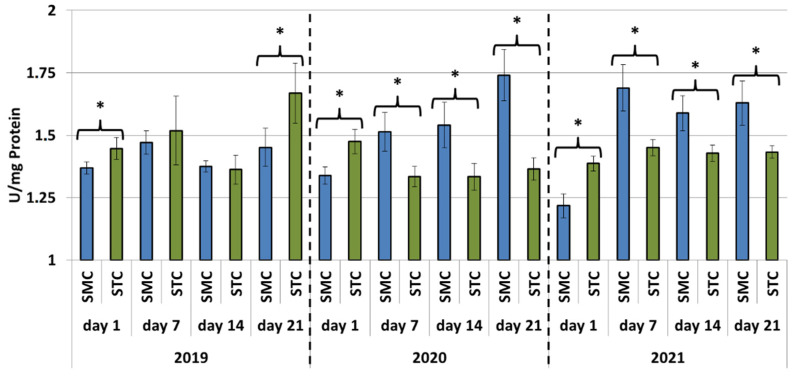
Alkaline protease activities in the hemolymph of workers in three consecutive years. SMC—workers reared in small-cell combs; STC—workers reared in standard-cell combs; *—differences between SMC and STC within the age group are significant at *p* ≤ 0.05.

**Figure 5:**
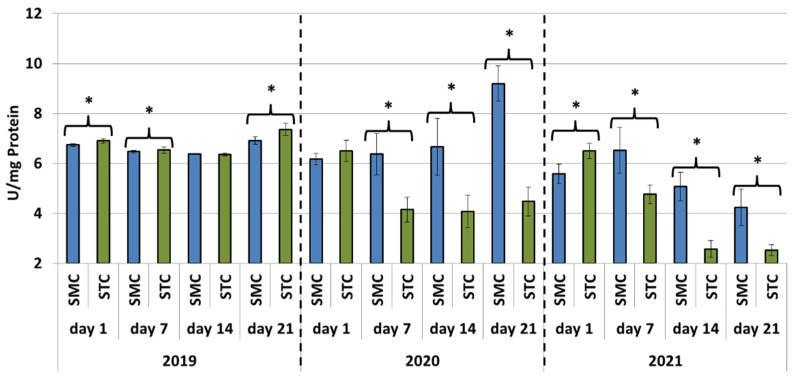
Acidic protease inhibitor activities in the hemolymph of workers in three consecutive years. SMC—workers reared in small-cell combs; STC—workers reared in standard-cell combs; *—differences between SMC and STC within the age group are significant at *p* ≤ 0.05.

**Figure 6:**
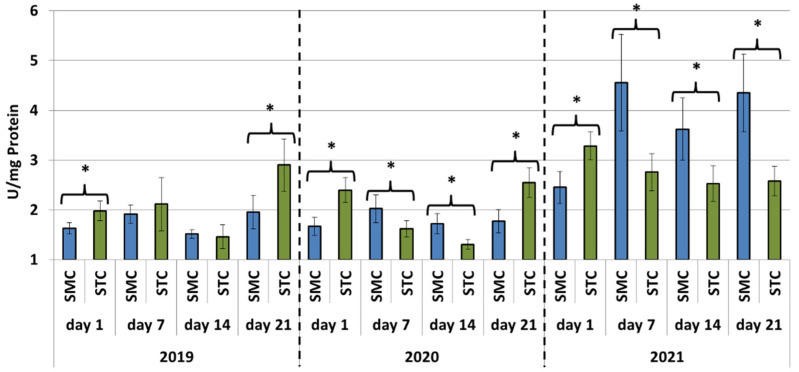
Neutral protease inhibitor activities in the hemolymph of workers in three consecutive years. SMC—workers reared in small-cell combs; STC—workers reared in standard-cell combs; *—differences between SMC and STC within the age group are significant at *p* ≤ 0.05.

**Figure 7:**
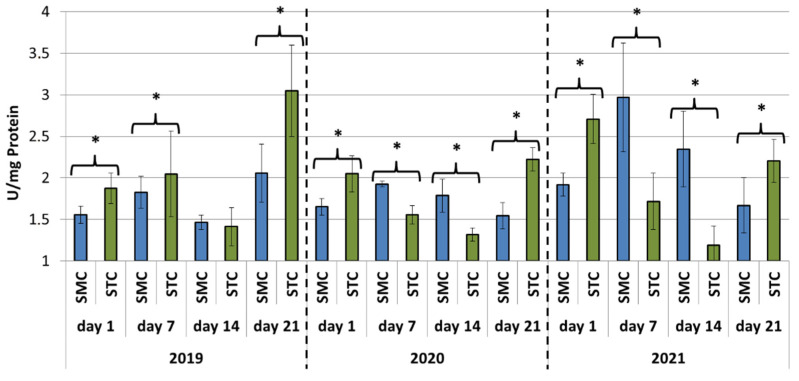
Alkaline protease inhibitor activities in the hemolymph of workers in three consecutive years. SMC—workers reared in small-cell combs; STC—workers reared in standard-cell combs; *—differences between SMC and STC within the age group are significant at *p* ≤ 0.05.

In all years (2019, 2020 and 2021), the protein concentrations in the 1-day-old bees were always significantly higher (*p* ≤ 0.05) in the SMC than STC workers (Figure 1). The opposite was found for the activities of proteases and their inhibitors, as it was always higher in the STC than SMC workers, and the difference was usually significant (*p* ≤ 0.05) (Figure 2, Figure 3, Figure 4, Figure 5, Figure 6 and Figure 7). The only exception were the activities of acidic protease inhibitors in 2020, i.e., the difference in this parameter between the STC and SMC workers was not statistically significant (Figure 5) (*p* = 0.12).

In the other age groups (7 d, 14 d and 21 d), the trends in the protein concentration (Figure 1) and in the activities of proteases (Figure 2, Figure 3 and Figure 4) and their inhibitors (Figure 5, Figure 6 and Figure 7) were always almost identical in 2020 and 2021. Regardless of the workers’ age (7 d, 14 d and 21 d), the protein concentrations were always significantly higher (*p* ≤ 0.05) in STC than in SMC (Figure 1). In turn, opposite results were shown for the activities of proteases and their inhibitors, i.e., they were significantly higher (*p* ≤ 0.05) in SMC than in STC (Figure 2, Figure 3, Figure 4, Figure 5, Figure 6 and Figure 7), with the exception of the activities of neutral protease inhibitors in the 21-day-old workers in 2020 (Figure 6) and alkaline protease inhibitors in the 21-day-old workers in 2020 and 2021 (Figure 7), which were significantly higher (*p* ≤ 0.05) in STC than in SMC.

In 2019, the trends in the values obtained in the groups of the 7-, 14- and 21-day-old workers differed significantly from those recorded in 2020 and 2021 (Figure 1, Figure 2, Figure 3, Figure 4, Figure 5, Figure 6 and Figure 7). The values of the hemolymph parameters differed significantly between the age groups. Additionally, one age group exhibited higher values in the SMC workers, another age group was characterized by higher values in the STC workers, or the values in the SMC and STC workers did not differ significantly.

In 2020, the activities of proteases in the age groups (7, 14 and 21 d) increased in SMC and remained at a similar level in STC (Figure 2, Figure 3 and Figure 4). In 2021, similar values of the parameter were recorded in both the SMC and STC workers, regardless of their age (Figure 2, Figure 3 and Figure 4). In contrast, no such homogeneous trends were noted in the values of protease inhibitors (Figure 5, Figure 6 and Figure 7).

### 3.2. Comb Cell Width

The width of the small-cell and standard-cell experimental combs did not differ between years (H = 2.935, df = 2, *p* = 0.230; H = 4.409, df = 2, *p* = 0.110, respectively; Kruskal–Wallis test).

The width of the small-cell experimental combs was significantly smaller (in 2019 *p* ≤ 0.01, *n* = 100; in 2020 *p* ≤ 0.01, *n* = 100; in 2021 *p* ≤ 0.01, *n* = 100; Mann–Whitney test) than that of the standard-cell experimental combs. The mean values of the width of the small-cell experimental combs reached 4.97 mm (SD = 0.051) in 2019, 4.96 mm (SD = 0.037) in 2020, and 4.95 mm (SD = 0.038) in 2021. The mean values of the width of the standard-cell experimental combs were 5.57 mm (SD = 0.056) in 2019, 5.56 mm (SD = 0.052) in 2020, and 5.57 mm (SD = 0.054) in 2021.

## 4. Discussion

As in the case of hygienic behavior [37,39] or bee lifespan [38], the width of comb cells in which workers were reared had a significant effect on the protein concentrations and the activities of proteases and their inhibitors in hemolymph.

The decrease in the protein concentration in hemolymph is correlated with age [46] and different tasks fulfilled by workers (nurse or forager) [47]. The highest protein concentrations were determined in larvae and pupae; these values were lower in nurse workers and the lowest in foragers [47,48]. In the present study, the width of the comb cells (small or standard) where the workers were reared had a significant impact on the protein concentrations in the 1-day-old workers. In all study years, it was significantly higher (usually *p* ≤ 0.01) in the SMC than STC workers. This may have been related to the nutrition of larvae in the small-cell combs reared by workers from standard-cell combs, as workers with larger body sizes are better feeders than smaller ones [49]. This was indirectly confirmed in our previous research showing that workers reared in standard-cell combs have a significantly larger head (width and height) than those reared in small-cell combs [36], which may be associated with the size and performance of hypopharyngeal glands. This hypothesis is worth confirming. Another cause of the significantly higher protein concentrations in the 1-day-old SMC workers may be the earlier activation of the proteolytic system in the STC than SMC workers, probably at the final pupal stage, through utilization of some portion of the protein. This is supported by the higher activities of proteases and their inhibitors on the first d of life in the STC workers.

Interestingly, the trends in the protein concentrations in the older workers (7, 14 and 21 d) were opposite to those in the 1-day olds. The values of this parameter were always significantly higher (*p* ≤ 0.01) in STC than in SMC (2020 and 2021), whereas the opposite was usually found in the case of the activities of proteases and their inhibitors. This may have been related to the predisposition of workers reared in the combs with the different cell widths (small or standard) to undertake different tasks in the colony. Since it has been reported that foragers are characterized by lower protein concentrations than nurse workers [47,48,50,51], it can be assumed that SMC workers more often serve as foragers and STC workers more often work in the nest, e.g., as nurse workers. As suggested by Crailsheim [50], nurse workers have a more efficient digestive system than foragers; hence, their bodies [52] and hemolymph [50] contain higher protein levels. Additionally, the digestive enzymes of nurse workers operate at a constant temperature prevailing in the brood rearing area in the nest, while the enzymes of foragers operate at different, often lower, temperatures. Foragers consume small amounts of pollen (protein) only to meet their needs. In turn, the protein concentration and pollen consumption by nurse workers depend on the nitrogen balance in the nest [52]. The publications cited above [47,48,50,51,52] indicate that the protein concentration in the hemolymph is one of the physiological indicators distinguishing foragers from nurse workers.

The higher activities of proteases and their inhibitors in the SMC workers may be associated with the higher exposure of foragers to pathogens. It can therefore be assumed that the width of comb cells determines the division of labor within the worker caste. This hypothesis was proposed in one of our earlier studies [36]. We assumed that, in addition to age polyethism, the significant variability in the comb cell width in nests formed without the wax foundation [29] introduces elements of morphological polyethism common to some ant species into the bee colony [53], which may represent a compromise between specialization and behavioral flexibility. Certainly, this hypothesis needs to be confirmed, but its validity may be supported by the research on bumblebees conducted by Spaethe and Weidenmüller [54] and Worden [55]. As reported by the authors, compared to small bumblebee workers, large individuals are more likely to work as foragers [54] and learn faster [55]. The present results indicate that the SMC workers had a greater predisposition to work as foragers, in contrast to the results obtained in the study of bumblebees. However, due to the significant differences in the social lifestyles between honeybees and bumblebees, these mechanisms may differ.

The results obtained in 2019 often differed significantly from those from the other two years. Probably, this did not result from the brood rearing conditions in the foster colonies, as the trends on the first day of workers’ life were similar in all the years. Differences were noted only in the older bees (7, 14 and 21 d). This may be related to the conditions in the colonies where the labeled bees were placed or is a consequence of environmental conditions. In our opinion, the prolonged period of severe drought in the time preceding the experiment in 2019 limited nectar flow of flower plants and in consequence limited the flight activities of foragers. The impact of many environmental factors on the foraging behavior has been confirmed by Abou-Shaara [56]. Therefore, to reduce the impact of accidental factors on the results of single-year research on honeybees, it is advisable that such investigations should be conducted for several consecutive years.

## 5. Conclusions

The width of comb cells where the workers were reared exerted a significant effect on the protein concentration and activities of proteases and their inhibitors in honeybee hemolymph. In the group of the 1-day-old workers, higher protein concentrations were determined in bees reared in the small-cell combs, whereas higher activities of proteases and their inhibitors were detected in individuals reared in the standard-cell combs. Opposite results were found in the older workers aged 7, 14, and 21 d.

The hypothesis of the effect of the width of comb cells where workers are reared on the division of labor in the colony is worth elucidation. This may explain the role of the high variability in the cell width in natural combs formed without the wax foundation and provide new knowledge of the evolution of the honeybee.

## Figures and Tables

**Table 1:** Number of hemolymph samples in each age group of workers (1 d, 7 d, 14 d and 21 d) in the three consecutive years.

	Sample Quantity							
Year	1 DAY		7 DAYS		14 DAYS		21 DAYS	
	SMC	STC	SMC	STC	SMC	STC	SMC	STC
2019	25	25	25	25	20	20	20	20
2020	10	10	20	20	20	20	20	20
2021	10	10	20	20	20	20	20	20

SMC—workers reared in small-cell combs; STC—workers reared in standard-cell combs.

**Table 2:** Values of the characteristics of weather conditions in years covering experimental period. The data are presented as average with ± standard deviation.

Year	Periods of Experiment (d)	Temperature Min (°C)	Temperature Max (°C)	Relative Humidity (%)	Rainy Days (per Period)	Average Rainfall (mm/m^2^ per Rainy d)
2019	1–7	10.14 ± 2.05	20.22 ± 1.48	68.29 ± 9.5	5	0.4
	8–14	10.64 ± 1.94	21.64 ± 1.64	66.43 ± 7.9	2	0.2
	15–21	13.22 ± 2.82	25.43 ± 2.66	68.28 ± 6.8	2	2.5
2020	1–7	15.71 ± 1.13	24.43 ± 3.18	84.57 ± 8.0	5	4.6
	8–14	15.21 ± 2.0	25.0 ± 2.28	77.3 ± 8.8	3	8.7
	15–21	13.0 ± 2.76	22.5 ± 4.33	76.3 ± 4.6	4	1.8
2021	1–7	10.66 ± 2.5	23.43 ± 1.32	61.43 ± 3.9	1	0.2
	8–14	11.0 ± 1.25	22.21 ± 2.84	72.14 ± 8.2	4	8.1
	15–21	15.57 ± 2.66	28.79 ± 1.66	65.9 ± 7.0	1	0.2

**Table 3:** Effect of the year (2019, 2020 and 2021) and age effect (1 d, 7 d, 14 d and 21 d) on hemolymph parameters of worker bees reared in small- and standard-cell combs.

Hemolymph Parameters		Impact of the Year		Impact of the Age					
				2019		2020		2021	
		SMC	STC	SMC	STC	SMC	STC	SMC	STC
proteins concentrations	H	110.718	17.971	59.814	63.748	35.397	24.488	39.526	24.126
	df	2	2	3	3	3	3	3	3
	*p*	0.000	0.000	0.000	0.000	0.000	0.000	0.000	0.000
activities of acidic proteases	H	68.288	40.181	65.198	65.936	38.954	30.565	28.372	29.851
	df	2	2	3	3	3	3	3	3
	*p*	0.000	0.000	0.000	0.000	0.000	0.000	0.000	0.000
activities of neutral proteases	H	58.011	84.145	65.952	63.674	27.363	31.819	28.135	31.621
	df	2	2	3	3	3	3	3	3
	*p*	0.000	0.000	0.000	0.000	0.000	0.000	0.000	0.000
activities of alkaline proteases	H	83.719	46.03	65.754	64.485	37.637	29.649	36.193	20.078
	df	2	2	3	3	3	3	3	3
	*p*	0.000	0.000	0.000	0.000	0.000	0.000	0.000	0.000
activities of acidic protease inhibitors	H	106.543	120.807	95.042	97.125	36.897	31.139	48.024	54.35
	df	2	2	3	3	3	3	3	3
	*p*	0.000	0.000	0.000	0.000	0.000	0.000	0.000	0.000
activities of neutral protease inhibitors	H	161.465	60.822	62.598	70.97	18.718	58.403	19.406	23.842
	df	2	2	3	3	3	3	3	3
	*p*	0.000	0.000	0.000	0.000	0.000	0.000	0.000	0.000
activities of alkaline protease inhibitors	H	44.443	6.086	73.821	73.873	42.088	59.088	43.027	54.529
	df	2	2	3	3	3	3	3	3
	*p*	0.000	0.048	0.000	0.000	0.000	0.000	0.000	0.000

SMC—workers reared in small-cell combs; STC—workers reared in standard-cell combs, H—value of the Kruskal–Wallis test, df—number of degrees of freedom, *p*—probability value.

## Data Availability

The data presented in this study are available on request from the corresponding author.
